# A Guide to the Practical Examination of Urine for the Use of Physicians and Students

**Published:** 1883-03

**Authors:** 


**A Guide to the Practical Examination of Urine for the Use of Physicians and Students.** By JAMES TYSON, M. D., Professor of General Pathology and Morbid Anatomy in the University of Pennsylvania; President of the Pathological Society of Philadelphia, etc., etc. Fourth edition; revised and corrected, with colored plates and wood engravings. Philadelphia: P. Blakiston, Son & Co., No. 1012 Walnut Street. 1883.

This is a book which has well deserved to reach a fourth edition. We have only to say of it, what we have said before, that it is one of the best working guides for the examination of urine extant. This last edition has been improved by the correction of some typographical errors, the introduction of some new illustrations, and a handsome lithographic copy of Vogel's Scale of Urine Tint.

